# Metabolic reprogramming‐driven homologous recombination and TCA cycle dysregulation contribute to poor prognoses in lung adenocarcinoma

**DOI:** 10.1111/jcmm.18406

**Published:** 2024-05-31

**Authors:** Zhanyu Xu, Dongming He, Liuliu Huang, Kun Deng, Wei Jiang, Junqi Qin, Zhiwen Zheng, Tiaozhan Zheng, Shikang Li

**Affiliations:** ^1^ Department of Thoracic and Cardiovascular Surgery The First Affiliated Hospital of Guangxi Medical University Nanning China

**Keywords:** homologous recombination, lung adenocarcinoma, metabolic reprogramming, SHFM1, tricarboxylic acid cycle

## Abstract

Increasing evidence has shown that homologous recombination (HR) and metabolic reprogramming are essential for cellular homeostasis. These two processes are independent as well as closely intertwined. Nevertheless, they have rarely been reported in lung adenocarcinoma (LUAD). We analysed the genomic, immune microenvironment and metabolic microenvironment features under different HR activity states. Using cell cycle, EDU and cell invasion assays, we determined the impacts of si‐SHFM1 on the LUAD cell cycle, proliferation and invasion. The levels of isocitrate dehydrogenase (IDH) and α‐ketoglutarate dehydrogenase (α‐KGDH) were determined by ELISA in the NC and si‐SHFM1 groups of A549 cells. Finally, cell samples were used to extract metabolites for HPIC‐MS/MS to analyse central carbon metabolism. We found that high HR activity was associated with a poor prognosis in LUAD, and HR was an independent prognostic factor for TCGA‐LUAD patients. Moreover, LUAD samples with a high HR activity presented low immune infiltration levels, a high degree of genomic instability, a good response status to immune checkpoint blockade therapy and a high degree of drug sensitivity. The si‐SHFM1 group presented a significantly higher proportion of cells in the G0/G1 phase, lower levels of DNA replication, and significantly lower levels of cell migration and both TCA enzymes. Our current results indicated that there is a strong correlation between HR and the TCA cycle in LUAD. The TCA cycle can promote SHFM1‐mediated HR in LUAD, raising their activities, which can finally result in a poor prognosis and impair immunotherapeutic efficacy.

## 1 INTRODUCTION

Non‐small cell lung cancer (NSCLC) is the most prevalent form of lung cancer (85%), and lung adenocarcinoma (LUAD) is the most prevalent type of NSCLC.^1^ Despite the continued use of emerging targeted therapies or immune checkpoint blockade in LUAD, patients with advanced disease are still prone to metastasis, resulting in an insignificant increase in their 5‐year survival rates.^2^ Therefore, the development of innovative therapeutic strategies based on new targets or signalling pathways is crucial. In recent years, treatments that target metabolism or DNA repair systems have reached clinical practice, demonstrating the value of targeting these pathways in cancer.^3^, ^4^, ^5^ Additionally, further research regarding the connections between metabolism and DNA repair pathways can lead to novel treatment strategies.

In normal human cells, DNA repair and metabolic pathways are essential for cellular equilibrium. During cancer, both pathways undergo significant alterations, including modifications that promote fast growth, genetic variability and survival.^6^, ^7^, ^8^ While these two fields of study have remained mostly separate, current evidence has suggested that these pathways are interconnected and intimately intertwined. The homologous recombination (HR) repair, which uses sister chromatids with homologous sequences as templates, is a high‐fidelity type of DNA double‐strand break repair that is most active in the S and G2 stages of the cell cycle. Additionally, genomic stability requires the maintenance of HR regulation.^9^ Furthermore, the metabolism of cancer cells regulates the development of vascular and circulatory systems, oxygen concentration and nutritional energy supply, requiring the interrelation of numerous oncogenes, growth factors and transcription factors (TFs), as well as reactive oxygen species (ROS).^10^ Several studies have suggested that the glycolytic pathway, which produces metabolites that are critical in DNA metabolism, might also be involved in preserving genomic integrity.^11^, ^12^ Despite several HR investigations, the therapeutic value of metabolic reprogramming in the development of separate high/low HR states remains unclear.

Various cancers have a poor prognosis when tumours experience metabolic reprogramming.^13^ Cancer cell metabolic reprogramming also affects the tumour immune microenvironment,^14^ and the immune response to anticancer therapy is impacted by the altered immunological microenvironment.^15^ Hence, understanding the mechanisms of metabolic reprogramming in HR states, as well as their implications for the survival of LUAD patients, is crucial. Herein, we investigated the therapeutic significance of common or unique biological characteristics that mediate HR states. First, we explored the patient's prognosis, immune infiltration, tumour stemness and genomic mutational status in different HR state groups in LUAD. Then, we evaluated the relationship between metabolic reprogramming and the HR activity status. Further, we used single‐cell RNA sequencing (scRNA‐seq) and showed that the HR activity in tumour cells was tightly related to metabolic reprogramming. Additionally, we found that the metabolic reprogramming gene SHFM1 plays a key role in LUAD, and its knockdown can influence the function of A549 cells and the TCA cycle. Finally, our current findings can give crucial support for treatment decisions regarding LUAD.

## 2 MATERIALS AND METHODS

### 2.1 Datasets and bioinformatics analyses

The RNA‐seq, somatic mutation and clinical data of lung cancer patients were obtained from The Cancer Genome Atlas (TCGA; http://cancergenome.nih.gov/) and five Gene Expression Omnibus (GEO; https://www.ncbi.nlm.nih.gov/) datasets (GSE31210, GSE50081, GSE37745, GSE68465 and GSE72094). GEO datasets were included if the following criteria were met: the source of the dataset samples was NSCLC or LUAD tissue specimens, each of which had a clear pathological diagnosis, and the dataset type was microarray or high‐throughput sequencing data. Exclusion criteria: data analysis required the removal of all data in each dataset except for LUAD. Based on the median HR activities, LUAD samples were categorized into high and low groups. Then, we used MAFTOOLS to compare the mutation profiles of both groups. RNA‐seq data were analysed for differential expression using the ‘Limma’ R package.^16^ The relationship between HR activity levels and overall survival (OS) of LUAD patients was analysed using the ‘survival’ R package. LUAD tissue specimens, each with a definite pathological diagnosis, and data from microarrays or high‐throughput sequencing platforms were the only dataset types considered from GEO. Data from each dataset that was not from LUAD patients were excluded before data processing.

### 2.2 Homologous recombination (HR) and metabolic reprogramming signature analysis

We used the LUAD RNA‐seq data to evaluate HR activity and metabolic reprogramming. HR was retrieved from the MSigDB (http://software.broadinstitute.org/gsea/msigdb). The ‘gene set variant enrichment analysis (GSVA)’ R package was used for enrichment analysis of gene sets from a single sample.^17^ The metabolic signature of gene sets previously described^18^ was retrieved, and each sample was analysed. This signature includes genes related to amino acid (348 genes), carbohydrate (286 genes) and integrated energy (110 genes) metabolism; lipids (766 genes), nucleotides (90 genes), tricarboxylic acid cycle (TCA cycle, 148 genes) and vitamin cofactor metabolism (168 genes). To evaluate score significance, *p*‐values were determined by the background distribution by permuting expression profiles (10,000,000 times).

### 2.3 Immune cell profiling analysis

Further, we investigated whether there was a connection between the levels of HR activity and tumour purity and immunological scores. Additionally, we evaluated whether there was a connection between gene expression and the immune infiltration of these immune cell types. To determine HR activity levels in immune cells, the ‘CIBERSORT’ and ‘ESTIMATE’ algorithms were applied to TCGA data to investigate high and low immune groups using a median cutoff. The CIBERSORT algorithm was selected for its precision in quantifying the composition of immune cells within the tumour milieu using gene expression data, enabling detailed immune profiling. The ESTIMATE method outputs estimated quantities of infiltrating stromal and immune cells, as well as predicted tumour purity, based on gene expression data.^19^ We applied the Microenvironment Cell Populations‐counter (MCP counter) method for its ability to accurately quantify the abundance of immune and stromal cell populations across various tissues, providing a comprehensive overview of the cellular landscape.^20^ Finally, the Tumour Immune Dysfunction and Exclusion (TIDE, http://tide.dfci.harvard.edu/) was chosen for its predictive power in assessing the likelihood of a patient's response to immunotherapy, linking immune infiltration patterns to potential treatment outcomes. It was employed to investigate the association between TAGAP expression and immunological cells, as well as to assess the probability of an immune response to immunotherapy.^21^

The single‐cell RNA‐seq data expression matrix (GSE189487) was retrieved from GEO. 6 LUAD cases yielded 16,487 scRNA‐seq samples. Then, we applied the ‘Seurat v. 4.0.4’ R package to analyse the expression matrix of single‐cell data. First, the ‘CreateSeuratObject’ program was used to examine the gene expression data, and the ‘Normalize Data’ program was applied for normalization. Next, the ‘FindVariableGenes’ tool was used to validate the highest 2000 variable genes. Subsequently, principal component analysis (PCA) was conducted via ‘RunPCA’. The useable PCs were found and aggregated using ‘FJackStraw’ and ‘Find Clusters’ at the greatest possible resolution. Finally, t‐SNE was used for visualization. Additionally, we used ‘Single R’ and ‘celldex’ R packages with ‘HumanPrimaryCellAtlasData’ for annotation. ‘Feature Plot’ and ‘Vln Plot’ were used to illustrate gene expression. In our study, we focused on evaluating the expression of the HR pathway across different pathological types by selecting key genes: NDUFA1, RPS5, RPS4Y1, BSG, RPS3, ODC1, OAZ1, ACADSB, NDUFAF3, CKB, PDK4, RPL13, FAU, NNMT and RPL10. These genes are crucial for maintaining genomic stability and repairing DNA double‐strand breaks, serving as essential components of the HR pathway.

### 2.4 Cell lines

The PC9, A549, Calu‐3 and NCI‐H1975 NSCLC cell lines and 16HBE (Human bronchial epithelial‐like cells) were supplied by the Type Culture Collection of the Chinese Academy of Sciences (Shanghai, China). Cells were incubated in RPMI‐1640 medium (Gibco, NY, USA) containing 10% fetal bovine serum (100 μg/mL streptomycin, 100 U/mL penicillin and 1.5 mg/L glutamine) at 37°C in a humidified incubator containing 5% saturated CO_2_.

### 2.5 Cell transfection

The si‐SHFM1 and si‐NC were transiently transfected into cells with Lipofectamine 3000 and cultured in the F12K medium, 5% CO_2_, 37°C incubators. Cells were cultured in a serum‐free medium for at least 24 h before transfection. Then, they were transfected for 24–48 h before subsequent experiments. The siRNA sequences are shown in Table 1.

**TABLE 1:** Primer and siRNA sequences.

id	Primer (5′‐3′)	
GAPDH	Forward	GCACCGTCAAGGCTGAGAAC
	Reverse	TGGTGAAGACGCCAGTGGA
SHFM1	Forward	ACGAGTTTGAAGAGTTCCCTG
	Reverse	ACCATGTTTCTCTAGTTCAGCTC
siRNA‐SHFM1		GCAGCCGGTAGACTTAGGTCTGTTA
siRNA‐NC		GCAGGCGATTCAGATCTGGTGCTTA

### 2.6 RNA isolation and qRT‐PCR

Total RNA was extracted from cells using TRIzol. The RNA concentration and purity (OD_260_/OD_280_) were evaluated using a micro‐nucleic acid detector. One microgram of total RNA was extracted and cDNA was generated using the Hifair® II First Strand cDNA Synthesis Kit. The expression levels of different genes were assessed using quantitative PCR with UltraSYBR Mixture, and the findings were quantified and normalized using $2^{-\Delta \Delta C_{t}}$ values. The primer sequences are described in Table 1.

### 2.7 Cell cycle analysis

After digestion with EDTA‐free trypsin, the cells were centrifuged at 2000 rpm for 5 min, rinsed with PBS, pre‐cooled, centrifuged again, and then mixed with the stain (UNICORD Cell cycle staining Kit 70‐CCS012). After 30 min, light‐suppressing flow cytometry was used to record the cell cycle distribution. The flow cytometry software was used to process the results.

### 2.8 EDU assay

Cells in the logarithmic growth phase were inoculated in six‐well plates lined with coverslips and transfected for 24 h. Then, the EDU staining was performed (refer to Reebok Cell‐Light EdU Apollo567 In Vitro Kit C10310‐1), followed by photographic analysis using a fluorescence microscope (200×).

### 2.9 Cell invasion assay

A total of 600 μL of complete medium was added to each chamber of 24‐well plates. Trypsin was used to digest cells for 24 h. The digestion was stopped with complete media, diluted to 50,000 cells/mL with a basal medium by centrifugation at 1000 rpm for 5 min, and then placed in the upper chamber. After incubation for 24 h, cells were fixed, stained, dried, blocked and photographed using a microscope (100×) and counted for analysis.

### 2.10 Detection of isocitrate dehydrogenase (IDH) and α‐ketoglutarate dehydrogenase (α‐KGDH)

The levels of α‐KGDH and IDH in si‐NC and si‐SHFM1 A549 cells were determined using quantification kits following the manufacturer's instructions (BioVision, Milpitas, CA, United States).

### 2.11 Central carbon metabolic profiling

The SHMF1‐knockdown A549 cell samples (*n* = 3) were used for metabolite extraction and then subjected to HPIC‐MS/MS analysis. For high‐performance ion exchange liquid chromatography (HPIC) separation, a Thermo Scientific Dionex ICS‐6000 HPIC System was employed (Thermo Fisher Scientific, IL, USA). We used an ESI interface‐equipped AB SCIEX 6500 QTRAP + triple quadrupole mass spectrometer (AB Sciex, USA) for assay formulation.

### 2.12 Statistical analyses

The associations between TAGAP mRNA expression and different score categories in the LUAD dataset were evaluated using the Pearson or Spearman correlation coefficients. To bolster the rigour of our findings, we conducted a thorough examination of an extensive array of clinical characteristics, which included, but were not limited to, age, sex, smoking status and tumour stage. These variables were scrutinized for their potential influence on patient prognosis and were systematically incorporated into our multivariate analyses. In our Cox regression model, adjustments were made for these factors to counteract possible confounding influences, ensuring the precision and reliability of our results. Cox regression analysis^22^ was used to explore whether clinical characteristics were associated with the prognosis of patients with LUAD. Survival rates were calculated using the Kaplan–Meier method, and comparisons among groups were performed using the log‐rank test. For each tumour, the Wilcoxon test was performed to analyse the expression variations between normal and cancerous samples. GraphPad Prism 5 software (GraphPad Software, San Diego, CA, USA) was utilized to analyse the data. Differences between groups were evaluated using one‐way ANOVA, Student's *t*‐test, and the chi‐square test. A *p* < 0.05 was considered statistically significant.

## 3 RESULTS

### 3.1 High HR activity is associated with poor prognosis in LUAD

**FIGURE 1:**
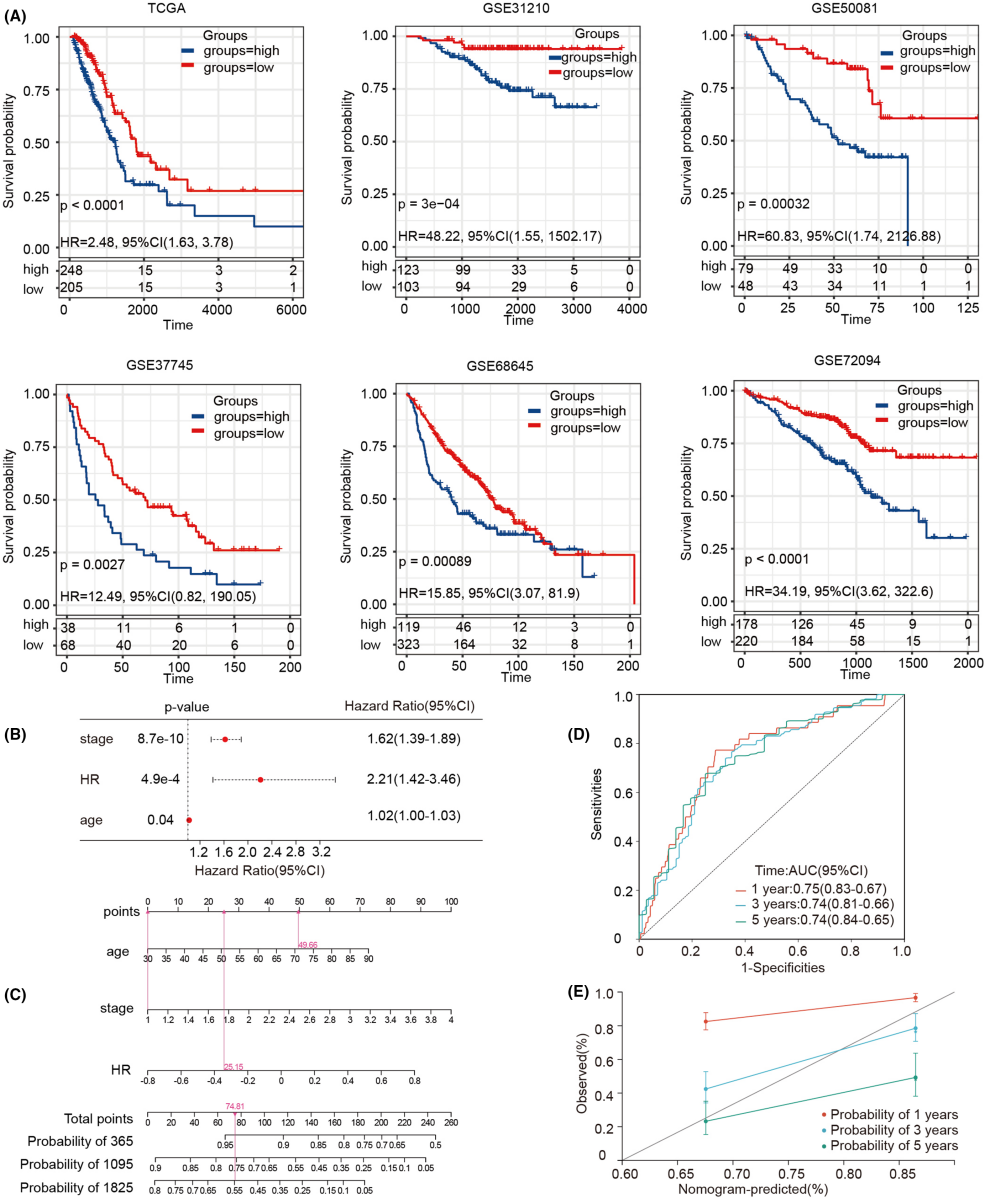
Effects of different HR activities on the survival of LUAD patients. (A) Kaplan–Meier plots of high and low HR activity in different LUAD cohorts (TCGA, GSE31210, GSE50081, GSE37745, GSE68465 and GSE72094) showed poor prognosis in the high HR activity group of LUAD. (B) Multivariate Cox regression analysis for different clinical features and HR activity. (C) Nomogram of TCGA‐LUAD patients based on age, stage and HR activity (red arrow shows an example). (D) The ROC curves are used to predict the overall survival (OS) for periods of 1, 3 and 5 years. (E) Calibration curves are generated to predict the overall survival (OS) for periods of 1, 3 and 5 years.

First, we used GSVA to investigate the prognostic significance of different HR activities in LUAD. Based on the median HR score in the TCGA and 5 GEO datasets (GSE31210, GSE50081, GSE37745, GSE68465 and GSE72094), we categorized samples into high and low‐HR groups. We found that high HR activity groups in LUAD had a poor prognosis (Figure 1A). A multivariate stepwise Cox regression analysis was conducted to evaluate multiple clinical and pathological features (Table 2), and we found that HR activity, stage and age were independent prognostic factors for TCGA‐LUAD patients (Figure 1B). These three variables were included in a multivariable Cox risk prediction model to obtain a risk prediction score for each patient, and the pattern was displayed using a nomogram, as shown in Figure 1C The overall C‐index of the model was 0.70 (95% CI: 0.65–0.75, *p* = 4.05e‐15). Time‐dependent ROC curves were plotted at year 1, year 3 and year 5, with an area under the curve of 0.765, 0.785 and 0.747, respectively, indicating good model accuracy (Figure 1D). Finally, multiple random overall samples were used to calibrate the curve (Figure 1E), and the results showed that the prediction curve fit well with the ideal curve, indicating that the prediction model did not suffer from overfitting.

**TABLE 2:** Clinical data and pathological parameters of TCGA‐LUAD patients in this study.

Variable	Overall, *N* = 446	Stage I, *N* = 240	Stage II, *N* = 108	Stage III, *N* = 74	Stage IV, *N* = 24	*p*‐Value
Age	66 (59, 72)	67 (59, 73)	65 (59, 72)	67 (58, 73)	62 (52, 69)	0.4
Gender	204 (46%)	97 (40%)	61 (56%)	33 (45%)	13 (54%)	0.037
HR	−0.06 (−0.42, 0.30)	−0.19 (−0.48, 0.20)	0.06 (−0.38, 0.34)	0.11 (−0.21, 0.39)	0.15 (−0.19, 0.39)	<0.001

### 3.2 High HR activity is associated with a low degree of immune infiltration, high genomic instability, good response status to immune checkpoint blockade (ICB) and high clinical drug sensitivity in LUAD

**FIGURE 2:**
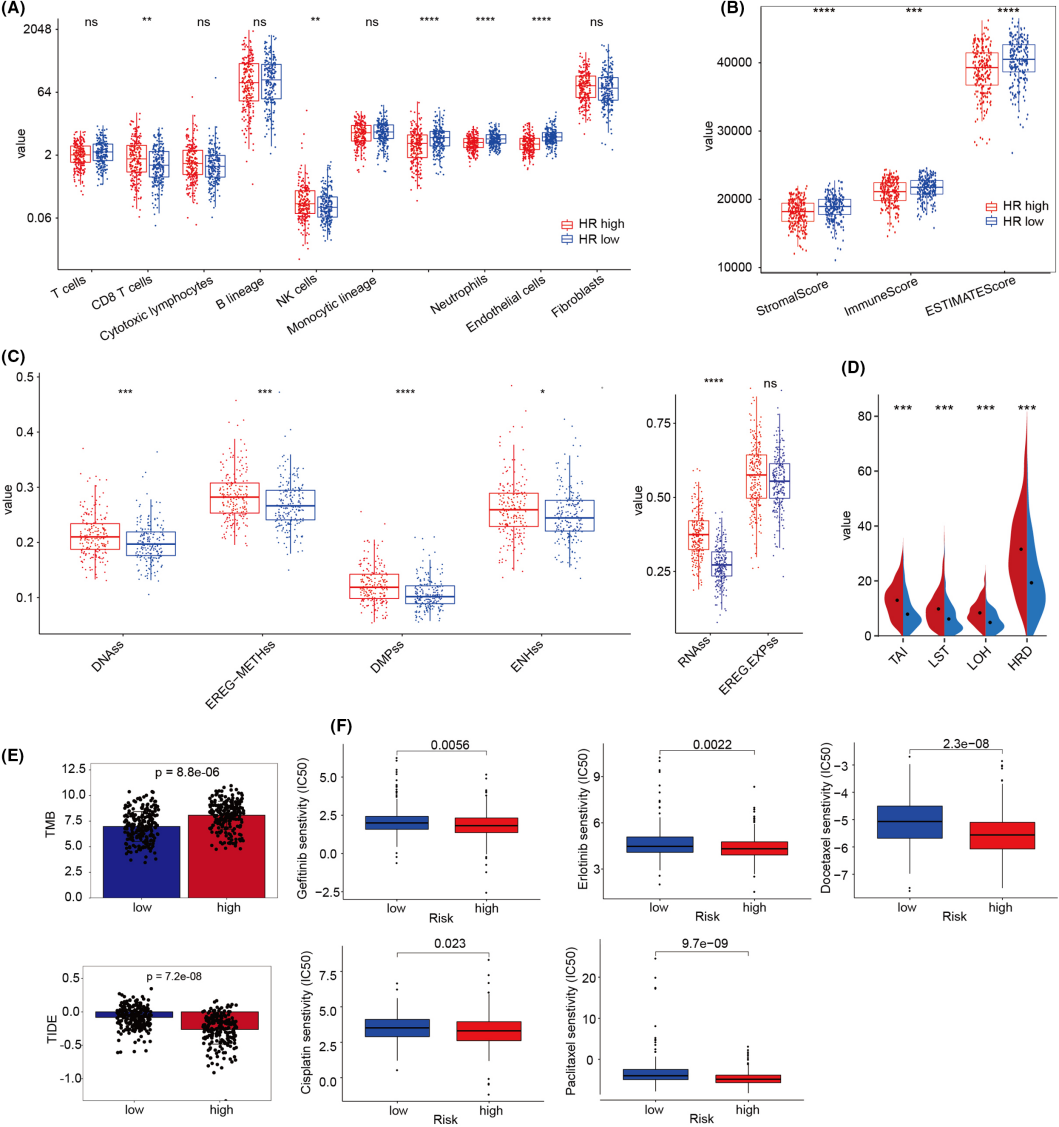
Characterization of the immune microenvironment and treatment responsiveness with different HR activities revealed that LUAD with high HR activity had a low degree of immune infiltration, a high degree of genomic instability, a good response status to ICB treatment and a high degree of drug sensitivity. (A) The boxplots present MCPcounter abundance estimates for eight immune cell species and two stromal cell populations in the TCGA‐LUDA cohort. (B) ESTIMATE, stromal and immune scores of TCGA‐LUAD patients by the ESTIMATE algorithm. (C–E) Boxplots of differences in DNA stemness scores, RNA stemness scores, HRD scores, TMB and TIDE scores in different HR activity groups. (F) IC_50_ values are shown for each anticancer drug in different HR activity groups. Statistical significance was determined by the Wilcoxon test. n.s, no significance, *p < 0.05, **p < 0.01, ***p < 0.001, ****p < 0.0001.

The MCPcounter and ESTIMATE algorithms were used to determine the immune infiltration of high and low HR LUAD samples. In the high HR group, we detected higher infiltration of NK and CD8 T cells but reduced infiltration of myeloid dendritic cells, neutrophils, and endothelial cells (Figure 2A). Additionally, the immune score, stromal score and total ESTIMATE score of this group were lower compared to the low‐HR group (Figure 2B). Next, we used 68 immuno‐signature scores to assess the differences between high and low‐HR groups (Figure S1A). Among them, 52 scores differed; 18 were elevated in the high HR group.

The RNA stemness (RNAss) score depends on mRNA expression, and DNA stemness (DNAss) depends on DNA methylation patterns, and both can be used to determine tumour stemness. In the high‐HR group, both RNAss and DNAss were greater than in the low‐HR group (Figure 2C). The homologous recombination deficiencies (HRD) score is composed of three scores: (1) the HRD‐loss of heterozygosity (HRD‐LOH), (2) the telomeric allelic imbalance (tAI) and (3) the large‐scale state transitions (LST), which reflect genomic instability at various levels. The high‐HR group had significantly higher LOH, LST, TAI and total HRD scores, thereby indicating a higher level of genomic instability (Figure 2D).

Immune checkpoint blockade (ICB) therapy has a better chance of being effective in patients who have a high tumour mutation burden (TMB).^23^ The TMB comprehends the total amount of somatic mutations in the genome of tumour cells. Patients with a high TMB can generate many neoantigens that stimulate antitumor immune responses.^24^ Herein, the TMB was significantly greater in the high‐HR group than in the low‐HR group (Figure 2E). Furthermore, TIDE is a gene expression biomarker developed to anticipate the clinical responsiveness to ICB therapy.^21^ TIDE refers to immunological malfunction and rejection caused by tumours. Patients with a higher TIDE score are more likely to experience anti‐tumour immune escape, leading to decreased ICB therapy response rates. In the present study, the TIDE scores were lower in the high‐HR group compared to the low‐HR group (Figure 2E). This result indicated a significant response rate to ICB therapy.

Moreover, we demonstrated that cells with HR deficiencies are vulnerable to numerous DNA‐damaging agents, and the high‐HR group was sensitive to various LUAD clinical agents, including gefitinib, erlotinib, cisplatin, paclitaxel and docetaxel, which had lower IC_50_ values in the high‐HR group (Figure 2F). These findings indicated that high‐HR activity in LUAD has a low degree of immune infiltration, a high degree of genomic instability and a good response status to ICB treatment as well as a high degree of drug sensitivity.

### 3.3 Genome landscape of different HR activities

**FIGURE 3:**
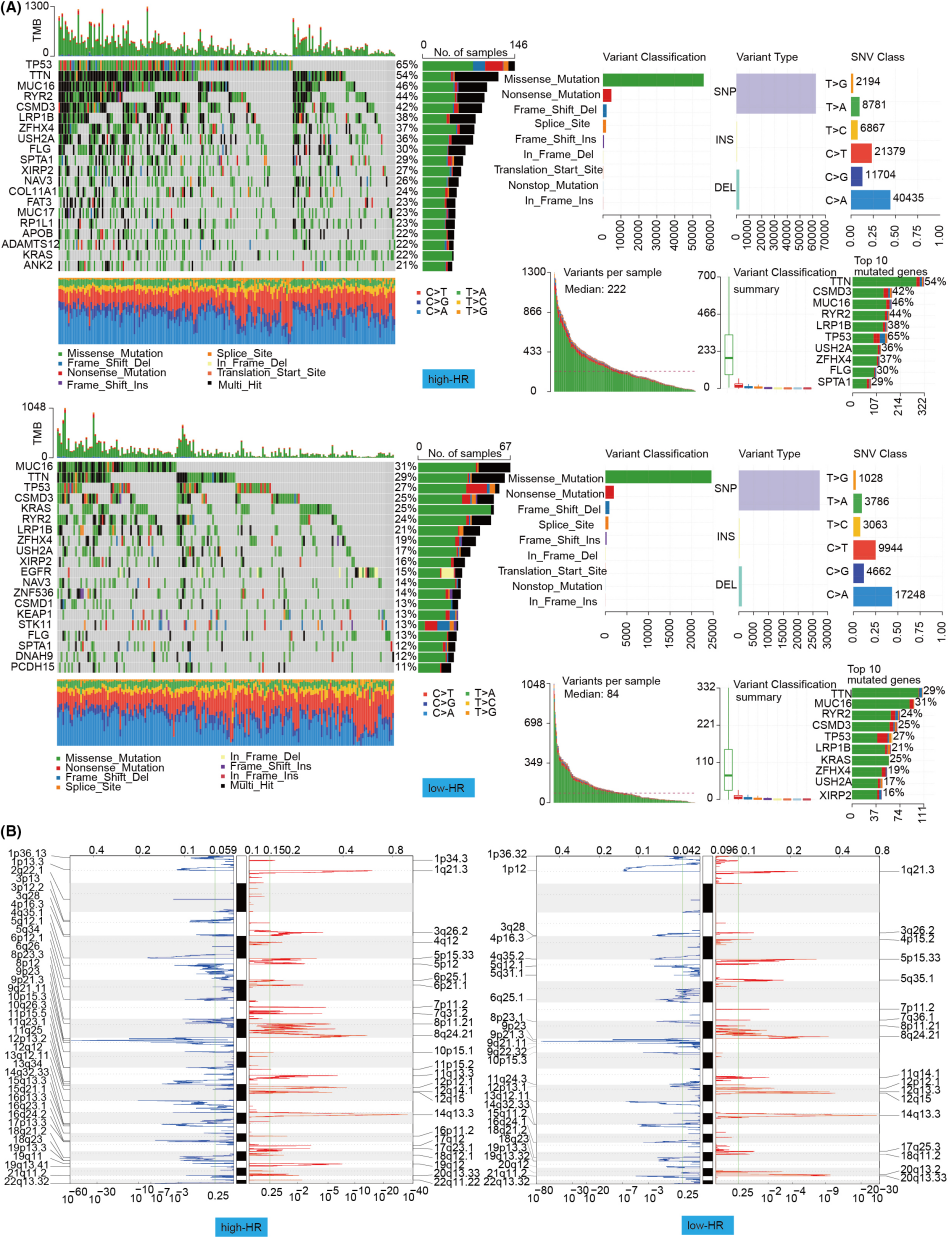
Genomic mapping of genetic alterations and copy number variants in different HR activity groups of TCGA‐LUAD. (A) Waterfall plot of the top 20 mutated genes with different HR activity in the TCGA‐LUAD cohort, mutation overview. (B) Gains and Losses of CNV in different HR activities of TCGA‐LUAD. The G‐score is at the top, and the *q*‐value is at the bottom.

Next, we retrieved single nucleotide mutation data for high and low HR patients from the TCGA database and used waterfall plots to depict mutation data for the top 20 genes in both patient groups (Figure 3A). Most alterations were missense mutations. Single nucleotide polymorphisms were more common than insertions and deletions, and C>A was the most prevalent single nucleotide variant. Then, we tallied the number of base mutations in each individual. The box plot displays the mutation type, and the horizontal histogram displays the features of the 10 leading mutations in high and low HR patients in descending order. Thus, we evaluated somatic copy number aberrations in high versus low HR patients (Figure 3B). High HR LUAD patients were repeatedly amplified at locus 1p34.3, 1q21.3, and 3q26.2 as well as 1p36.13, 1p13.3, and 2q22.1 had deletions. On the other hand, the low HR LUAD group was repeatedly amplified at locus 1q21.3, 3q26.2 and 4p15.2, as well as 1p36.32, 1p12, and 3q28 presented deletions.

### 3.4 Distinct metabolic reprogramming of HR activity is associated with prognosis in LUAD

**FIGURE 4:**
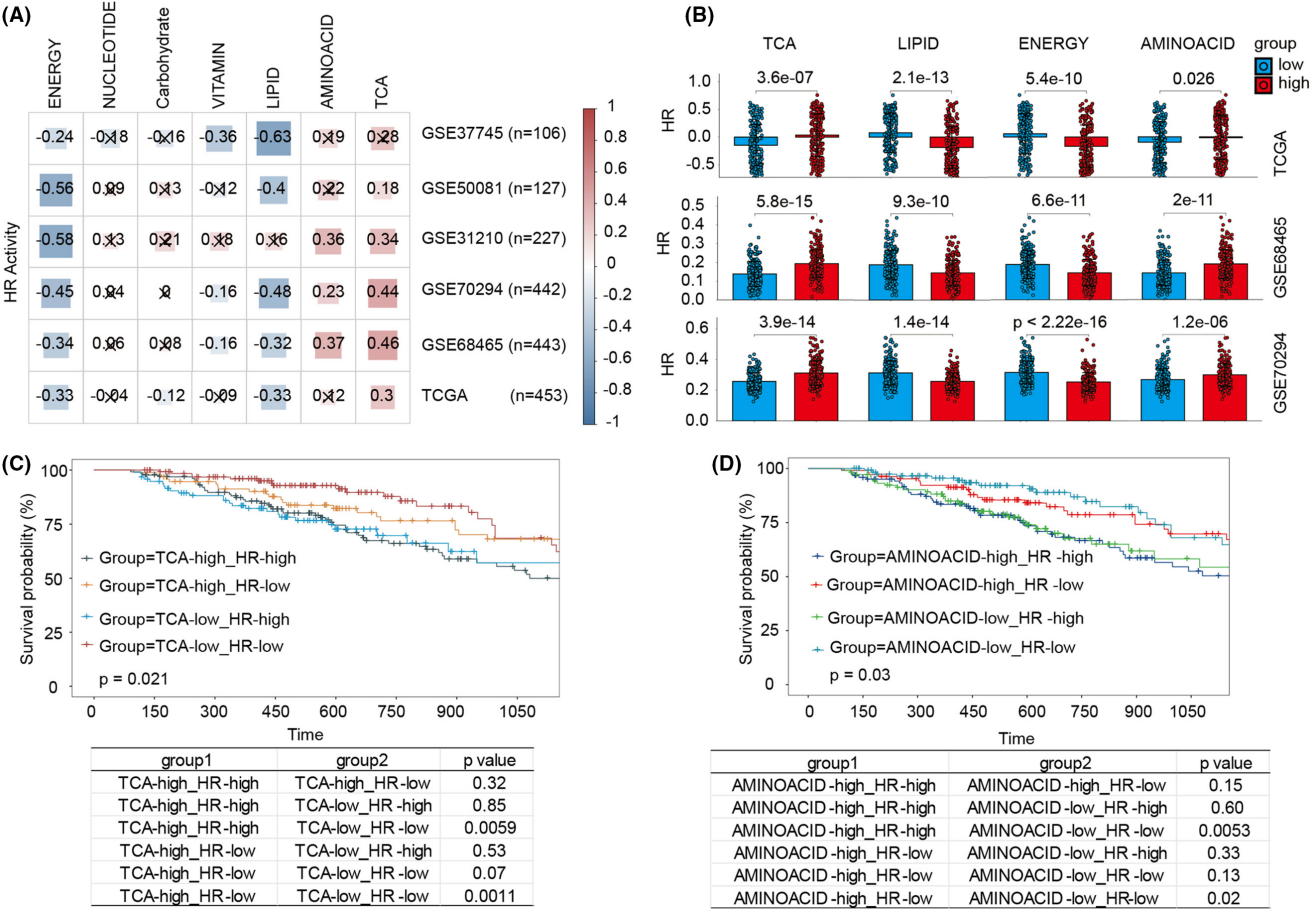
Relationship and prognostic impact of the molecular features of different metabolic reprogramming processes on the HR activity in LUAD. (A) Correlation of metabolic reprogramming with HR activity in the 6 LUAD cohorts. Cross icons (‘×’) represent data points where the Spearman correlation coefficient is not statistically significant. (B) Boxplots of the differential distribution of HR activity on different metabolic reprogramming subgroups in the TCGA‐LUAD, GSE68465 and GSE70294.Statistical significance was determined by the Wilcoxon test. (C) Survival curves showing the prognostic impact of different HR activities, TCA metabolic status and amino acid metabolic status on LUAD patients.

To explore the HR activity based on different metabolic reprogramming, we examined RNA‐seq data of LUAD from TCGA and examined their HR activity information. We used GSVA to evaluate the different metabolic reprogramming activities of LUAD. We found that HR activity varied for different metabolic reprogramming activities in LUAD. Notably, in all TCGA and GSE50081, GSE37745, GSE31210, GSE68465 and GSE72094 LUAD data cohorts, the energy and lipid metabolism were negatively correlated with HR activity, while TCA cycle and amino acid metabolism were positively correlated with HR activity (Figure 4A). Next, we examined the HR enrichment scores in LUAD, which returned seven metabolic features. In the TCGA and the two largest sample size datasets (GSE72094 and GSE68465 cohorts), HR activity was significantly enriched in the high TCA cycle and amino acid metabolism groups, but the opposite result was observed in the lipid and energy metabolism groups (Figure 4B). The group characterized by low TCA cycle activity and low HR activity (TCA‐low_HR‐low) exhibited a statistically significant improvement in survival compared to the high‐activity TCA and HR group (TCA‐high_HR‐high, *p* = 0.0059). Furthermore, this survival benefit was consistently observed when comparing the TCA‐low_HR‐low group to the group with high TCA cycle activity but low HR activity (TCA‐high_HR‐low, *p* = 0.0011, Figure 4C). In the context of amino acid metabolism, the group with low activity in both amino acid metabolism and HR (AMINOACID‐low_HR‐low) also demonstrated a significant survival advantage when compared to the high‐activity amino acid and HR group (AMINOACID‐high_HR‐high, p = 0.0053), and this trend was maintained in comparison with the group with high amino acid metabolism activity but low HR activity (AMINOACID‐high_HR‐low, *p* = 0.019, Figure 4D). Altogether, these findings revealed that metabolic reprogramming is not a straightforward operation incorporating cancer cell migration and invasion. Additionally, alterations to the complex tumour microenvironment may develop HR‐aggressive cancers.

### 3.5 Positive correlation between TCA cycle, amino acid metabolism and HR at the single‐cell transcriptional level

**FIGURE 5:**
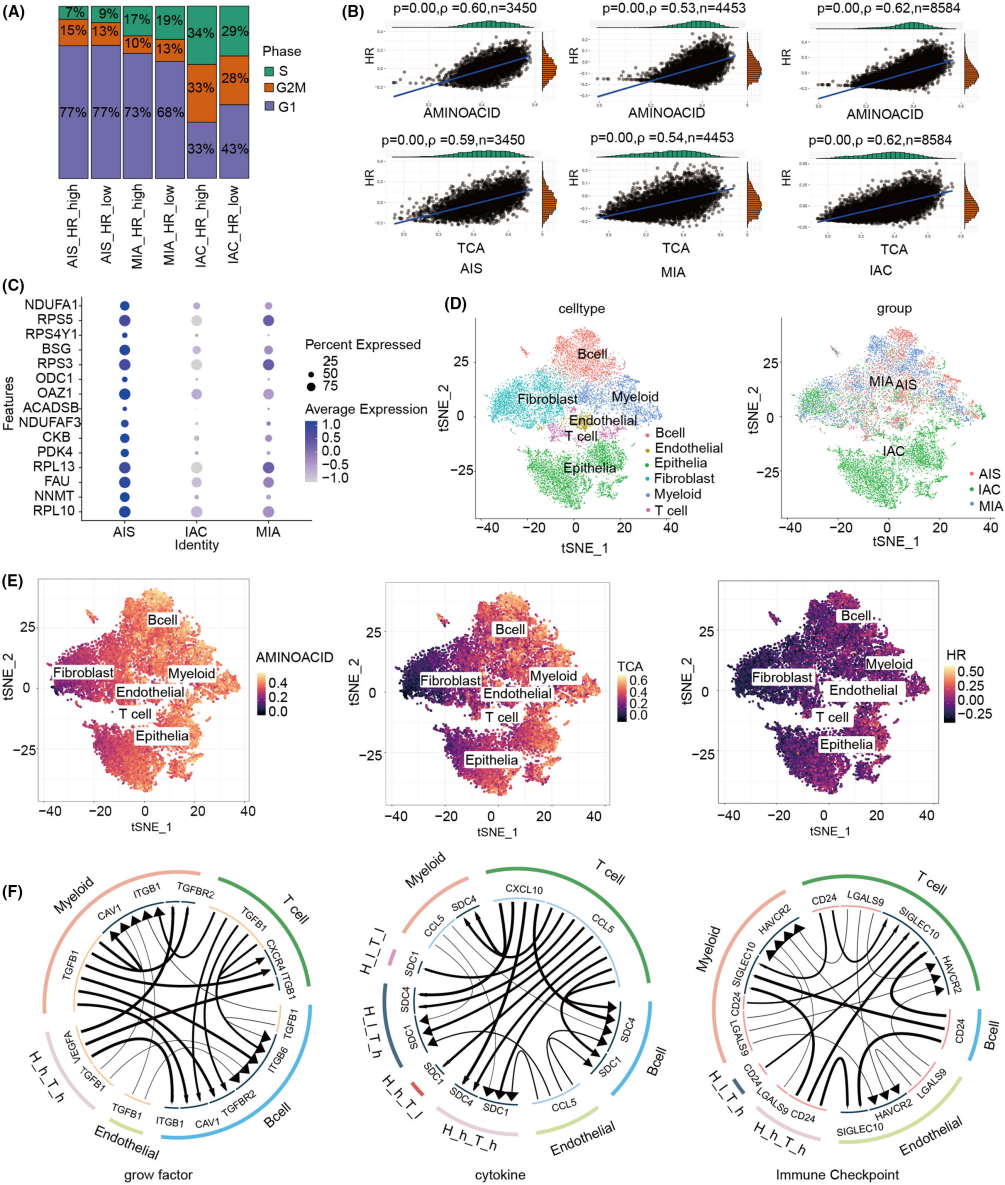
Positive correlations between the TCA cycle, amino acid metabolism and HR at the single‐cell transcriptional level. (A) Differences in cell cycle ratios of LUAD cells in different HR states in AIS, MIA and IAC groups. (B) Correlation of different HR states with TCA cycle and amino acid metabolism in LUAD cells of AIS, MIA and IAC groups. (C) The dot plots showed a strong correlation between the expression of HR markers and the expression of TCA cycle and amino acid metabolic markers in AIS, MIA and IAC. (D) The t‐SNE plot shows the clustering results and the different sources of cells, including AIS, MIA and IAC. (E) The t‐SNE plot shows the distribution of GSVA scores for the TCA cycle, amino acid metabolism and HR. (F) Analysis of cellular communication of different TCA HR groups with other cells and epithelial cells. H_h_T_h, HR_high‐TCA_high; H_h_T_l, HR_high‐TCA_low; H_l_T_h, HR_low‐TCA_high; H_l_T_l, HR_low‐TCA_low.

The scRNA‐seq data of GSE189487 were screened by strict quality control criteria and annotated for all cells. We obtained 16,487 cells from LUAD histology samples from AIS, MIA to IAC for our subsequent analysis. Next, we evaluated the HR and MR activity of all cells using GSVA. Based on the median HR values, we assessed the cell cycle profile of high and low HR in AIS, MIA and IAC. As LUAD pathology progressed, the number of cells in the G2M and S phases gradually increased in the high‐HR group (Figure 5A). Moreover, the positive correlation between HR activity and the TCA cycle and amino acid metabolism gradually increased (Figure 5B). Then, we examined markers of HR and TCA cycle, as well as amino acid metabolism in three LUAD samples. We used dot plots to illustrate the expression of the previously stated markers in the various original samples. The expression of HR markers in AIS and MIA was significantly related to the expression of TCA cycle and amino acid metabolism indicators (Figure 5C). Hence, these results demonstrated at the molecular marker level the relationship between HR and the TCA cycle, the metabolism of amino acids. Six cell clusters (B cell, endothelial, epithelia; fibroblast, myeloid and T cell) were identified using t‐SNE after gene filtering, normalization and PCA (Figure 5D). There are different degrees of activation of the HR, TCA cycle and amino acid metabolism in different cells. Additionally, the activation status of all three is evident in epithelial cells (Figure 5E).

Furthermore, we classified each cell into one of four groups according to its median score: HR‐high‐TCA‐high, HR‐high‐TCA‐low, HR‐low‐TCA‐high and HR‐low‐TCA‐low. To explore the distinctions between double high and low groups, we selected differentially expressed genes (DEGs) using Seurat's Findmarker function and performed GO and KEGG enrichment analyses. The enrichment analysis revealed that the humoral immune response, complement activation, immunoglobulin‐mediated immune response, B cell‐mediated immunity, complement activation classical pathway and humoral immune response mediated by circulating, phagosome and antigen processing and presentation were enriched (Figure S1B).

Then, we used iTALK to build a cellular communication network based on potential interactions between receptor‐ligand pairs to identify the molecular connections between epithelial cells and other types of cells. We analysed the cellular connections relying on the roles of receptor‐ligand combinations in three modules: Growth factors, cytokines and immune checkpoints (Figure 5F). We detected significant intercellular communication in the double‐high group in the growth factor module. The expression of VEGFA was elevated in the double‐high group compared to the other groups. Thus, cells in the double‐high group might employ the VEGFA‐ITGB1 axis to enhance tumour growth and metastasis.^25^ The Warburg effect and production of ROS were specifically boosted by TGFB1‐CAV1 activation in the double‐high group. In this case, the Warburg effect and ROS generation were enhanced, thereby increasing the potential of cancer metastasis.^26^ For example, in ovarian cancer, TGFB1‐ITGB6 can enhance metastasis, lipid metabolism and cisplatin resistance.^27^

Moreover, we found that the double‐high group had higher levels of SDC1 than the other cytokine module groups. High levels of SDC1 can lead to tumour cell proliferation, invasion and drug resistance, which are related to bad prognoses.^28^ In the immune checkpoint module, the double‐high group would activate the immune system more than the single‐high group, resulting in a particular interaction between cancer cells and immune cells mediated by CD24‐SIGLEC10.

### 3.6 The HR‐related gene SHMF1 has an important role in LUAD

**FIGURE 6:**
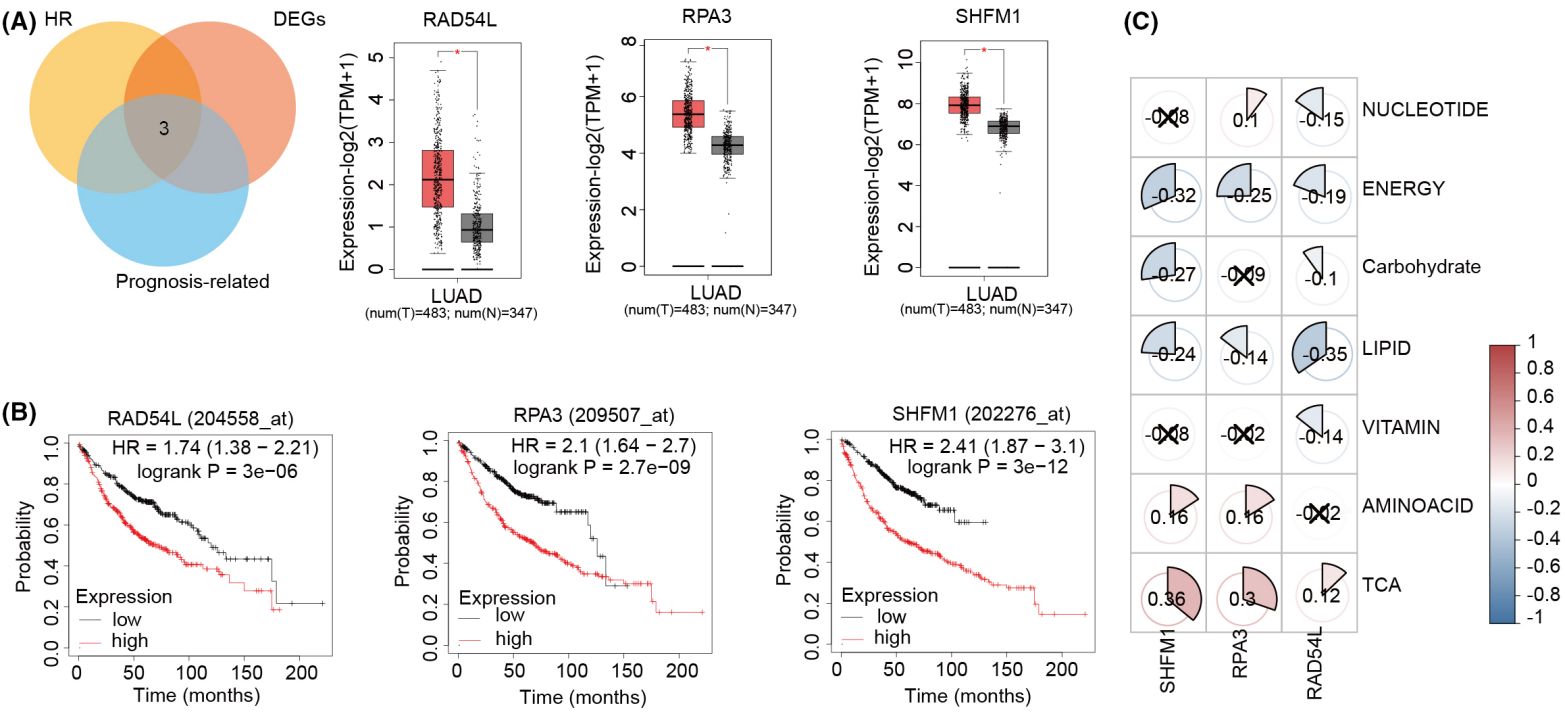
Prognostic and Expression Analysis of RAD54L, SHFM1 and RPA3 in Lung Adenocarcinoma. (A) Venn diagram of genes associated with HR, DEGs and LUAD prognosis and differential expression of RAD54L, SHFM1 and RPA3 in LUAD and paraneoplastic tissues by GEPIA. (B) Survival curves of RAD54L, SHFM1 and RPA3 in LUAD. (C) Heatmap of the correlation between RAD54L, SHFM1 and RPA3 and metabolic reprogramming. Cross icons (‘×’) represent data points where the Spearman correlation coefficient is not statistically significant. Statistical significance was determined by the Wilcoxon test. *p < 0.05.

Further, we investigated HR‐related genes. First, we screened their expression differences in LUAD and paraneoplastic tissues. Using GEPIA, we found that four of the 28 HR‐related genes—RAD51, RAD54L, RPA3 and SHFM1—were significantly highly expressed in LUAD tissues (Figure 6A). Then, we used KMplotter to evaluate the survival curves of the four genes. We found that elevated expressions of RAD54L, RPA3 and SHFM1 were associated with a poor prognosis for LUAD patients (Figure 6B). Finally, a comprehensive Spearman correlation analysis of these three genes and metabolic reprogramming activities revealed a significant positive correlation of SHMF1 with amino acid metabolism, TCA cycle and lipid metabolism in LUAD (Figure 6C).

### 3.7 Functional analysis of SHFM1 in LUAD cell lines

**FIGURE 7:**
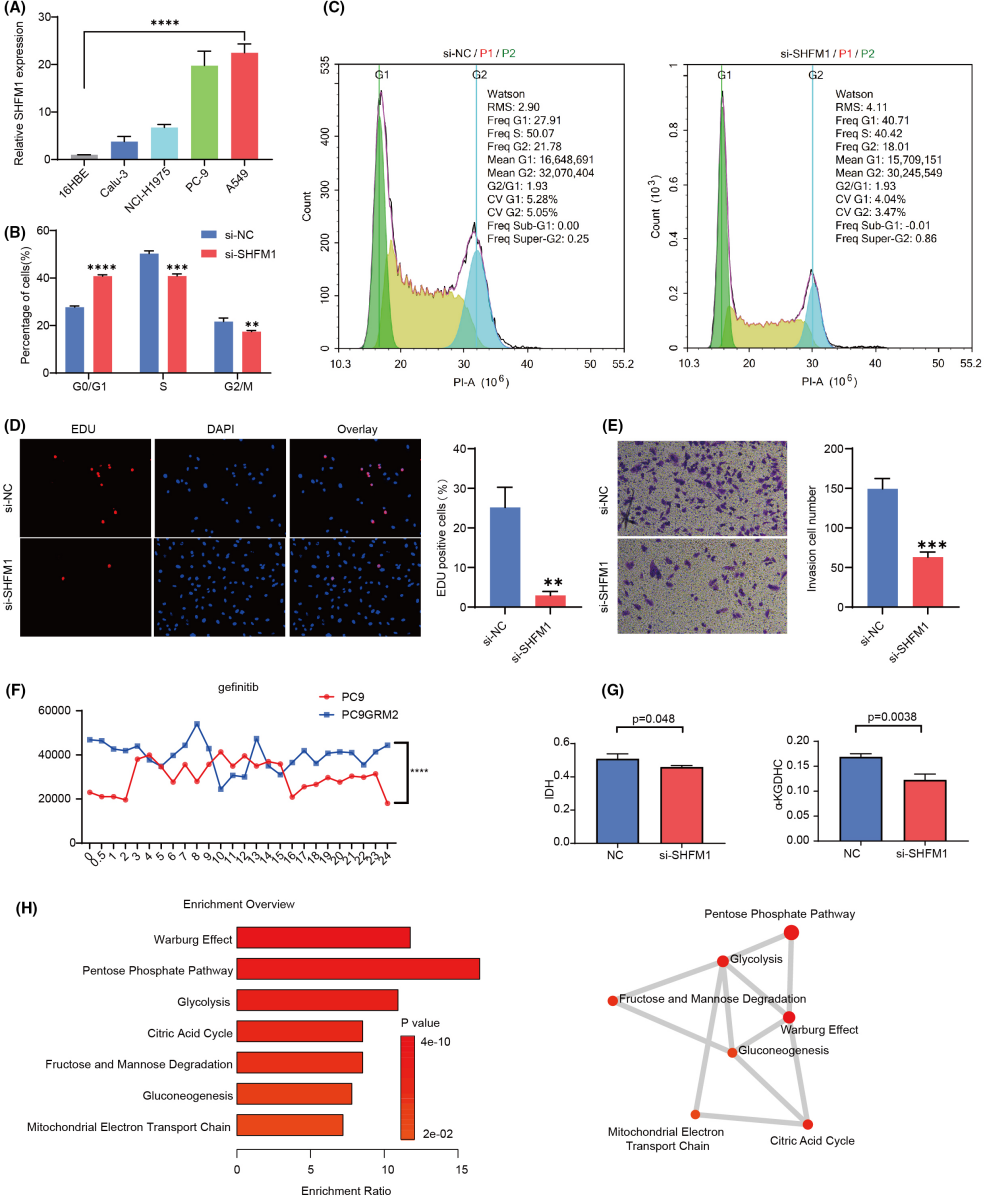
Exploring the mechanism of SHFM1 in A549 cell line. (A) Expression of SHFM1 in four LUAD cells and normal lung cells by qRT‐PCR. (B) The proportions of A549 cells transfected with si‐SHFM1 and si‐NC at different cell cycle phases. (C) Cell cycle of A549 cells transfected with si‐SHFM1 and si‐NC by flow cytometry. (D) Proliferation of A549 cells transfected with si‐SHFM1 and si‐NC by EDU assay. (E) Migration ability of A549 cells transfected with si‐SHFM1 and si‐NC by the transwell migration assay. (F) Temporal expression of SGFM1 in PC9 and drug‐resistant cell lines (PC9GRM2). (G) Concentrations of isocitrate dehydrogenase and α‐ketoglutarate dehydrogenase of si‐SHFM1 A549 cells by ELISA. (H) Functional enrichment of differentially expressed metabolites in si‐SHFM1 cells and entry action network map. Statistical significance was determined by unpaired, two‐tailed Student's t‐test. **0.005 <  *p* < 0.01; ***0.0001 <   *p*< 0.005; **** *p* < 0.0001.

The qRT‐PCR results indicated that the expression of SHFM1 was higher in the four types of LUAD cells compared to normal lung cells, and the highest expression was detected for A549 cells (Figure 7A). The flow cytometry results demonstrated that the proportion of cells in the G0/G1 phase was significantly higher in the si‐SHFM1 group than in the si‐NC group, while the number of cells in the S and G2/M phases was lower (Figure 7B,C). The EDU staining demonstrated that the si‐SHFM1 group had a lower level of DNA replication than the si‐NC group (Figure 7D). Additionally, the cell migration levels in the si‐SHFM1 group were much lower than in the si‐NC group, according to the cell migration assay (Figure 7E). Then, to investigate the role of SHFM1 on LUAD therapy response, we analysed the changes in SHFM1 expression over 24 h in gefitinib‐resistant (PC9GR) and gefitinib‐sensitive cell lines using the GSE34228 dataset (PC9). Within 24 h, we found that PC9GR‐resistant cell lines presented greatly enhanced SHFM1 expression levels (Figure 7F). These results suggested that SHFM1 might be associated with gefitinib resistance in LUAD. Next, we measured the concentrations of isocitrate dehydrogenase and α‐ketoglutarate dehydrogenase in si‐SHFM1 A549 cells using ELISA. We found that the levels of both TCA enzymes were significantly lower compared to the negative control (Figure 7G). Then, we determined 56 central carbon metabolism‐like target metabolites in si‐SHFM1 and NC groups of A549 cell samples by HPIC‐MRM‐MS/MS. The pathway enrichment for differential metabolites showed that the Warburg effect, pentose phosphate pathway, glycolysis, citric acid cycle, fructose and mannose degradation, gluconeogenesis and mitochondrial electron transport chain metabolic pathways were significantly enriched (Figure 7H). These results indicated that the TCA cycle might promote SHFM1‐mediated HR in LUAD.

## 4 DISCUSSION

In the cellular genome, several therapeutic modalities that target tumours, such as ionizing radiation from radiation therapy, cause DNA double‐strand breaks (DSBs). This DNA damage triggers intracellular pathways that either cause cancer cells to die or repair the damaged DNA.^29^ HR is one of the primary DSB repair processes and is, therefore, a potential cancer treatment target.^30^ Herein, patients with high HR activity had a poor prognosis across all LUAD cohorts, and HR was a standalone prognostic factor for TCGA‐LUAD patients. High HR activity in LUAD was also related to a low degree of immune infiltration, a high degree of genomic instability and a good response status to ICB treatment, as well as a high degree of drug sensitivity. Moreover, we found that the high‐HR group was more sensitive (lower IC_50_ values) to various LUAD clinical agents, including gefitinib, erlotinib, cisplatin, paclitaxel and docetaxel. We also demonstrated that HR‐defective cells are particularly susceptible to DNA‐damaging substances that can cause interstrand crosslinks (ICL), such as mitomycin C and cisplatin.^31^, ^32^ These findings have been recently used in clinical research on cancer treatment. ERCC1, an endonuclease implicated in nucleotide excision repair and homology‐directed repair, confers cisplatin resistance. Additionally, adjuvant cisplatin therapy can be beneficial for ERCC1‐negative NSCLC patients.^33^ The c‐Abl activation of Rad51, an important player in HR, imatinib, a c‐Abl kinase inhibitor, has also been studied as a sensitizer in DNA‐damaging therapy.^34^ Besides, the proteins involved in HR can repair DNA damage induced by other chemotherapeutic drugs such as camptothecin and gemcitabine.^35^, ^36^ Consistent with the findings of our study, in irradiated cells, Cyclin B1/CDK1 mitochondrial migration and mitochondrial ATP production were both improved at the same time as nuclear DNA repair, according to Lili Qin.^37^ A broad link between ionizing radiation‐induced changes in mitochondrial metabolic parameters and ionizing radiation‐induced DNA DSB repair kinetics was demonstrated by Adam Krysztofiak using statistical modelling of DNA repair and metabolic data.^38^ Overall, these results have demonstrated the significance of the HR machinery in cancer treatment.

Metabolic reprogramming and HR are key biological processes that are closely associated. However, their relationship has not been fully explored. Therefore, in the present study, we used scRNA‐seq to examine the positive correlation between metabolic reprogramming and HR at the single‐cell level. We found that the number of cells in the G2M and S phases gradually rose as LUAD developed in the high‐HR group, and the positive correlation between HR activity and the TCA cycle and amino acid metabolism gradually increased. Meanwhile, we detected different degrees of activation of HR and TCA cycle and amino acid metabolism in different cells, and the activation status of these three features was evident in epithelial cells. Related to our study, cancers with HRD require oxidative metabolism to produce NAD+ and ATP for poly (ADP‐ribose) polymerase (PARP)‐dependent DNA repair processes, as demonstrated by lvaro Lahiguera et al.^39^

Further, we investigated HR‐related genes and found that SHFM1 had a significant positive correlation with amino acid metabolism, TCA cycle and lipid metabolism in LUAD patients. Additionally, increased expression of SHFM1 was related to poor prognosis. SHFM1, also known as Deleted in Split Hand/Foot 1 (DSS1), was initially identified in patients with hereditary heterogeneous limb development disease and is referred to as split hand/split foot malformation type 1 (SHFM1) or external rectus muscle.^40^ SHFM1 binds to the longest conserved portion of the BRCA2 protein in mammalian cells and is essential for BRCA2 stability and function, thereby making a crucial contribution to the activity of BRCA2 in mediating HR.^41^ Through ubiquitin‐mediated protein hydrolysis, SHFM1 also controls other cellular processes, such as DNA repair. Multiple biological processes are related to SHFM1, such as development, differentiation, DNA repair, HR, genome stabilization, cell proliferation, tumour transformation, protein degradation and mRNA export.^42^ Herein, we showed that SHFM1 plays an important role in the metabolic reprogramming of LUAD. Besides, SHFM1 can also make HeLa cells more sensitive to cisplatin.^43^ Sanne et al.^43^ have explored the FA/BRCA pathway members and demonstrated that this pathway is important not only in the cisplatin response in head and neck SCC but also in other squamous cell carcinomas. They also found that the levels of SHFM1 are usually higher in HNSCC than in healthy mucosa and can greatly vary.^44^ Additionally, a recent investigation on ovarian cancer has shown that SHFM1 contributes to cisplatin resistance, with higher SHFM1 levels in tumour cells compared to nontumor surrounding cells. These results indicated provided the basis for using SHFM1 expression as a biomarker for ovarian cancer.^45^ Finally, high expression of SHFM1 is also associated with poor prognosis and recurrence of breast cancer‐free survival.^46^

In the present study, significantly more A549 cells entered the G0/G1 phase after treatment with si‐SHFM1, while fewer cells entered the S and G2/M phases, along with DNA replication and cell migration. Within 24 h, the expression levels of SHFM1 significantly increased in the PC9GR‐resistant cell line. The levels of isocitrate dehydrogenase and α‐ketoglutarate dehydrogenase in si‐SHFM1 A549 cells were significantly reduced compared to the negative control. Further, we determined 56 central carbon metabolism‐like target metabolites in si‐SHFM1 and NC groups of A549 cells by HPIC‐MRM‐MS/MS and found that the differential metabolites were significantly enriched for the Warburg effect, Pentose Phosphate Pathway and Glycolysis. Metabolic pathways such as citric acid cycle, fructose and mannose degradation, gluconeogenesis and mitochondrial electron transport chain. Finally, these results suggested that the TCA cycle might promote SHFM1‐mediated HR in LUAD.

In conclusion, we demonstrated the role of HR activity in LUAD using multi‐omics. We also investigated the impact of metabolic reprogramming on HR and found that their mutual promotion can result in malignant development and immunotherapy response.

## AUTHOR CONTRIBUTIONS

**Zhanyu Xu:** Conceptualization (lead); methodology (lead); writing – original draft (equal). **Dongming He:** Data curation (equal). **Liuliu Huang:** Validation (lead); writing – review and editing (lead). **Kun Deng:** Software (equal). **Wei Jiang:** Software (equal). **Junqi Qin:** Investigation (equal). **Zhiwen Zheng:** Data curation (equal). **Tiaozhan Zheng:** Writing – original draft (equal). **Shikang Li:** Funding acquisition (lead); supervision (lead).

## FUNDING INFORMATION

The Natural Science Foundation of Guangxi province (2022GXNSFAA035513), the Joint Project on Regional High‐Incidence Diseases Research of Guangxi Natural Science Foundation (2024GXNSFBA010032), the Youth Science Foundation of Guangxi Medical University (GXMUYSF202420), the National Key Clinical Specialty Construction Project and the Guangxi Key Clinical Specialty Construction Project funded this research.

## CONFLICT OF INTEREST STATEMENT

The authors confirm that there are no conflicts of interest.

## Supporting information


Figure S1.

## Data Availability

The study's original contributions are contained in the article/supplementary material. Any additional questions should be forwarded to the respective author.
