# Supplementary material for: Metabolic reprogramming‐driven homologous recombination and TCA cycle dysregulation contribute to poor prognoses in lung adenocarcinoma
Source: J Cell Mol Med. 2024 May 31;28(11):e18406. doi: 10.1111/jcmm.18406 (PMC11142899; doi:10.1111/jcmm.18406)
Supplement: Supplementary file 1 — Figure S1. [file JCMM-28-e18406-s001.doc]

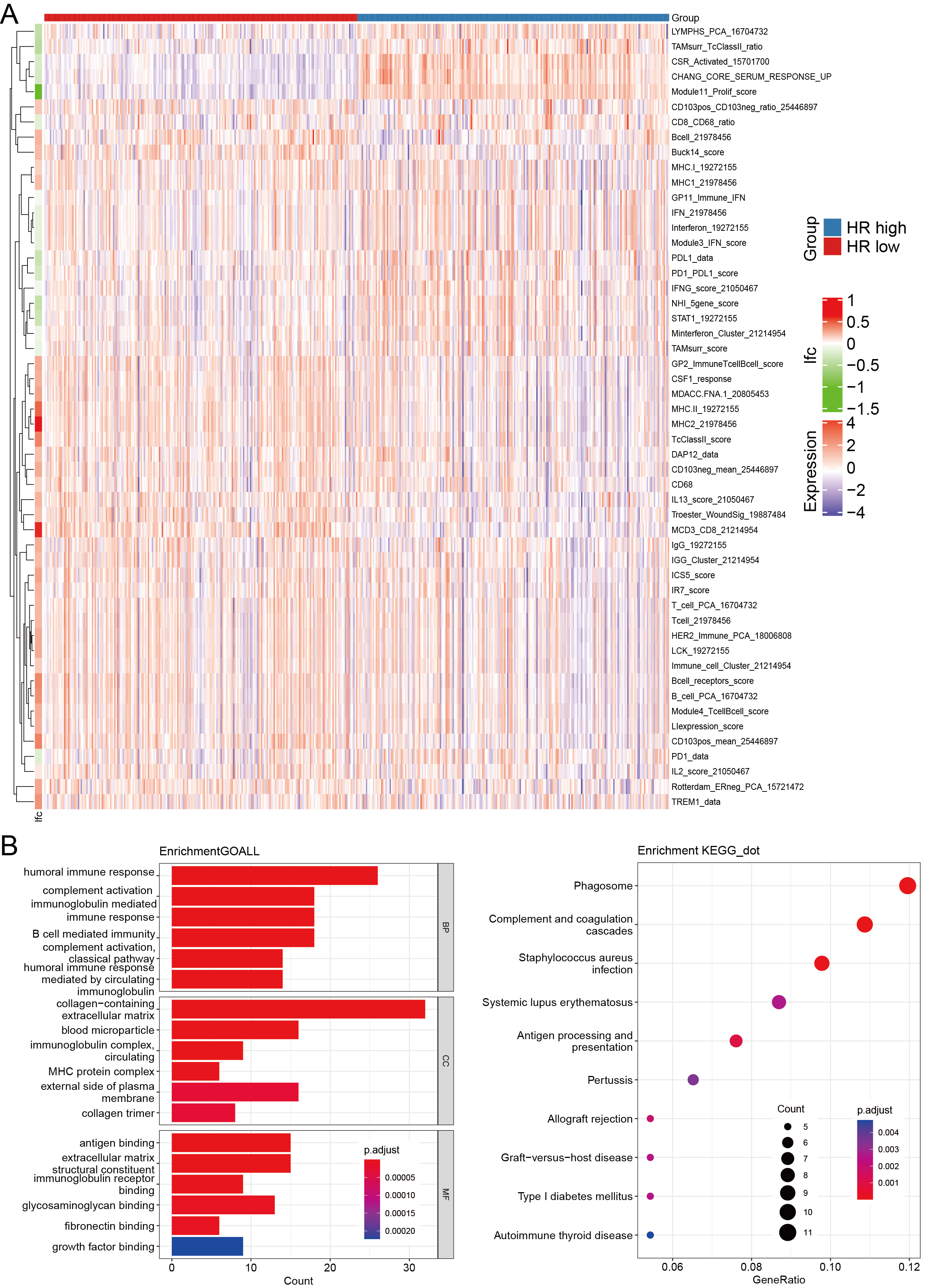


Figure S1.Differential Immune Signature Profiling and Functional Enrichment Analysis Across HR Activity Groups (A) Heatmap of 68 immune signatures at different HR activities. (B) GO and KEGG functional enrichment of DEGs in epithelial cells in the double-high group versus the remaining subgroups.
